# Extracellular vesicles in bone and periodontal regeneration: current and potential therapeutic applications

**DOI:** 10.1186/s13578-020-00527-8

**Published:** 2021-01-12

**Authors:** Leila Gholami, Vajihe Taghdiri Nooshabadi, Shiva Shahabi, Marzieh Jazayeri, Rana Tarzemany, Zohreh Afsartala, Khatereh Khorsandi

**Affiliations:** 1 Department of Periodontics, Dental Research Center, Hamadan University of Medical Sciences, Hamadan, Iran; 2 Department of Tissue Engineering and Applied Cell Sciences, School of Medicine, Semnan University of Medical Science, Semnan, Iran; 3 Nervous System Stem Cells Research Center, Semnan University of Medical Sciences, Semnan, Iran; 4 Student Research Committee, School of Dentistry, Hamadan University of Medical Sciences, Hamadan, Iran; 5 Department of Oral Biological and Medical Sciences, Faculty of Dentistry, The University of British Columbia, Vancouver, Canada; 6 Brain and Spinal Cord Injury Research Center, Neuroscience Institute, Tehran University of Medical Science, Tehran, Iran; 7 Department of Tissue Engineering and Applied Cell Sciences, School of Advanced Technologies in Medicine, Tehran University of Medical Sciences, Tehran, Iran; 8 Department of Photodynamic, Medical Laser Research Center, Yara Institute, ACECR, Tehran, Iran

**Keywords:** Extracellular vesicles, Mesenchymal stem cells, Bone regeneration, Periodontal regeneration

## Abstract

Oral mesenchymal stem cells (MSCs) and their secretomes are considered important factors in the field of medical tissue engineering and cell free biotherapy due to their ease of access, differentiation potential, and successful therapeutic outcomes. Extracellular vesicles (EVs) and the conditioned medium (CM) from MSCs are gaining more attraction as an alternative to cell-based therapies due to the less ethical issues involved, and their easier acquisition, preservation, long term storage, sterilization, and packaging. Bone and periodontal regenerative ability of EVs and CM have been the focus of some recent studies. In this review, we looked through currently available literature regarding MSCs’ EVs or conditioned medium and their general characteristics, function, and regenerative potentials. We will also review the novel applications in regenerating bone and periodontal defects.

## Introduction

Periodontitis is still considered as a globally prevalent disease [1]. The chronic presence of pathological factors may proceed to destruct the supporting periodontium of the teeth and lead to tooth loss. Early diagnosis of periodontitis prevents further structural damages to the periodontium, and it can be treated by removal of pathologic factors using scaling and root planning [2]. In the case of lost periodontal tissues, regeneration of the periodontium is considered as a challenging treatment. Numerous procedures and products have been developed and applied to regenerate lost periodontal tissue [3–7]. Such regenerative treatments are difficult and only effective in specific conditions with limited tissue reconstruction results, as the periodontium is a complex structure which possess various cell types [8].

Bone, as a connective tissue, preserves and supports organs and tissues within the body. It is also one of the important structures of the periodontal tissues surrounding teeth. Bone remodeling is a lifelong process to preserve bone structure and function. Some conditions like aging, trauma, obesity, congenital abnormalities, surgical removal of a mass within the bone, and cancer metastases to the bone, may interfere with the normal balance of bone remodeling and increase the demand for an efficient therapy to regenerate the bone tissue [9–12]. Autogenous and allogenous bone grafts are currently considered as a gold standard in bone regenerative therapies. However, numerous complications including, morbidity at graft harvesting site, limited harvesting sources, graft versus host disease (GVHD), need for secondary surgery, infection, and non-union formation are associated with these treatments [13–17]. Therefore, a new, safe, and efficient therapy is highly demanded to overcome the existing limitations. Bone remodeling involves various cells, such as bone cells (osteoblasts, osteoclasts, mechanosensitive osteocytes, and bone marrow stem cells), immune cells (T cells, dendritic cells, and monocytes), and articular cartilage cells [18]. Intercellular communication between cells is essential for bone remodeling [19]. This has directed recent studies towards investigating more suitable and efficient bone regenerative therapies especially when dealing with challenging defects that are beyond the spontaneously healing size.

Regenerative medicine is considered as a subdivision of translational medical science that focuses on identifying various approaches to efficiently replace or reestablish the normal structure and function of damaged tissues [20]. Stem cells have been considered as effective tools in regenerative medicine, with the potential to differentiate into various cell types, and having a wide range of applications including in tooth regeneration, wound healing, and treatment of various diseases [21, 22].

Oral tissues have been considered a suitable source of mesenchymal stem cells (MSCs), and the first dental derived stem cells were isolated from a dental pulp in 2000 [23]. Dental stem cells are regarded as an easily accessible and suitable source of stem cells with a well-known regenerative capacity. Dental derived stem cells include multiple types such as dental pulp mesenchymal stem cells (DP-MSCs), stem cells from exfoliated deciduous teeth (SHED), stem cells from apical papilla (SCAP), periodontal ligament stem cells (PDLSCs), and dental follicle progenitor cells (DFPCs) .There, still exists a search for finding more suitable stem cell origins in the oral cavity to be used in tissue regenerations and cell based therapies [24].

One of the secreted particles from MSCs is extracellular vesicle (EVs). EV is a term approved by International Society for Extracellular Vesicles (ISEV) for bilayer lipid membrane vesicles that are non-replicable, containing nucleic acids, proteins, lipids, and various signaling molecules [25]. Most eukaryotic cells secrete EVs, which have essential roles in intercellular communications. They carry active signals that can influence the activity of adjacent or distant recipient cells [26, 27]. It has been suggested that MSCs’ paracrine activity is controlled by growth factors and survival signals, as well as EVs. Current investigations have shown the beneficial contribution of MSC derived EVs in MSCs’ physiological functions [28]. Due to the challenges related to stem cell therapy, more recent studies have focused on other novel alternative regenerative methods such as cell free therapies on based paracrine signaling and use of such secreted particles to overcome these obstacles [29–33]. The investigation onstem cells and their mechanisms of action have revealed the important role of bioactive molecules of these cells and the media surrounding them [the conditioned media (CM)]. One of the most important secreted molecules that are released to the biological fluid or cell culture CM are EVs that show the same regenerative function as stem cells and can be considered as safe alternatives [34]. EVs’ valuable advantages over stem cell therapy are their relative ease of preservation and sterilization, and the capability of long-term storage without the risk of losing their properties. These cell-secreted particles provide broad bio-signaling functions for various targeted cell types.

This capability has attracted attention to use EVs for transferring particular messages to multiple heterogeneous cells involved in tissue regeneration therapies such as craniofacial bone and tissue regenerations. The current review aims to summarize the available evidence on EVs’ function and also their potential applications in bone and periodontal regeneration.

## General characteristics of EVs: biogenesis, components, and composition

**Fig. 1:**
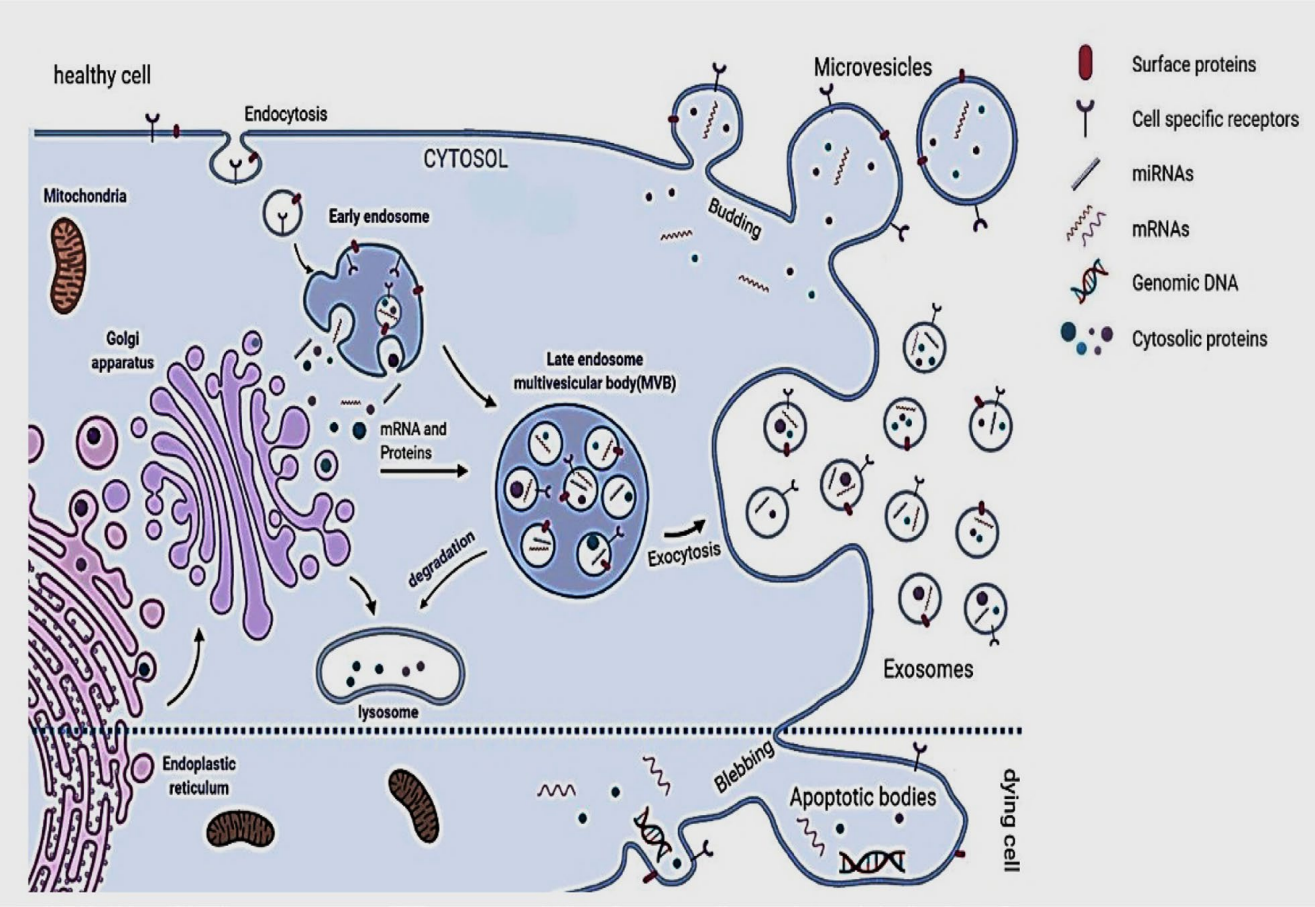
Mechanisms of maturation and secretion of extracellular vesicles

EVs have been previously classified into three main subtypes based on their cellular origin, size, or biogenesis. This includes (1) exosomes (30–150 nm) with an endocytic origin, (2) microvesicles (100–1000 nm) formed by budding of the plasma membrane, and (3) apoptotic bodies (500 nm–2 µm) derived from dying cells (Fig. 1) [35]. Based on new guidelines and the fact that determining the exact biogenesis pathway of EV is still considered difficult, use of a more general term of EV is recommended. Moreover, for identifying EV subtypes, use of more operational terms which refer to either their physical characteristics such as size, density, biochemical composition, descriptions of conditions or cell of origin is suggested [25, 36].

Exosomes were first recognized in 1981 [37] and can be distinguished from other EVs by their protein and lipid composition. They can be secreted from almost all cell types and they can be found in body fluids (e.g., blood, breast milk, saliva, semen, and urine) [38, 39].

Exosomes are formed by the inward budding of endosomal membranes of multivesicular Endosome (MVE) and form intraluminal vesicles (ILV). These exosomes are released due to the fusion of the MVE with the plasma membrane [35].

**Fig. 2:**
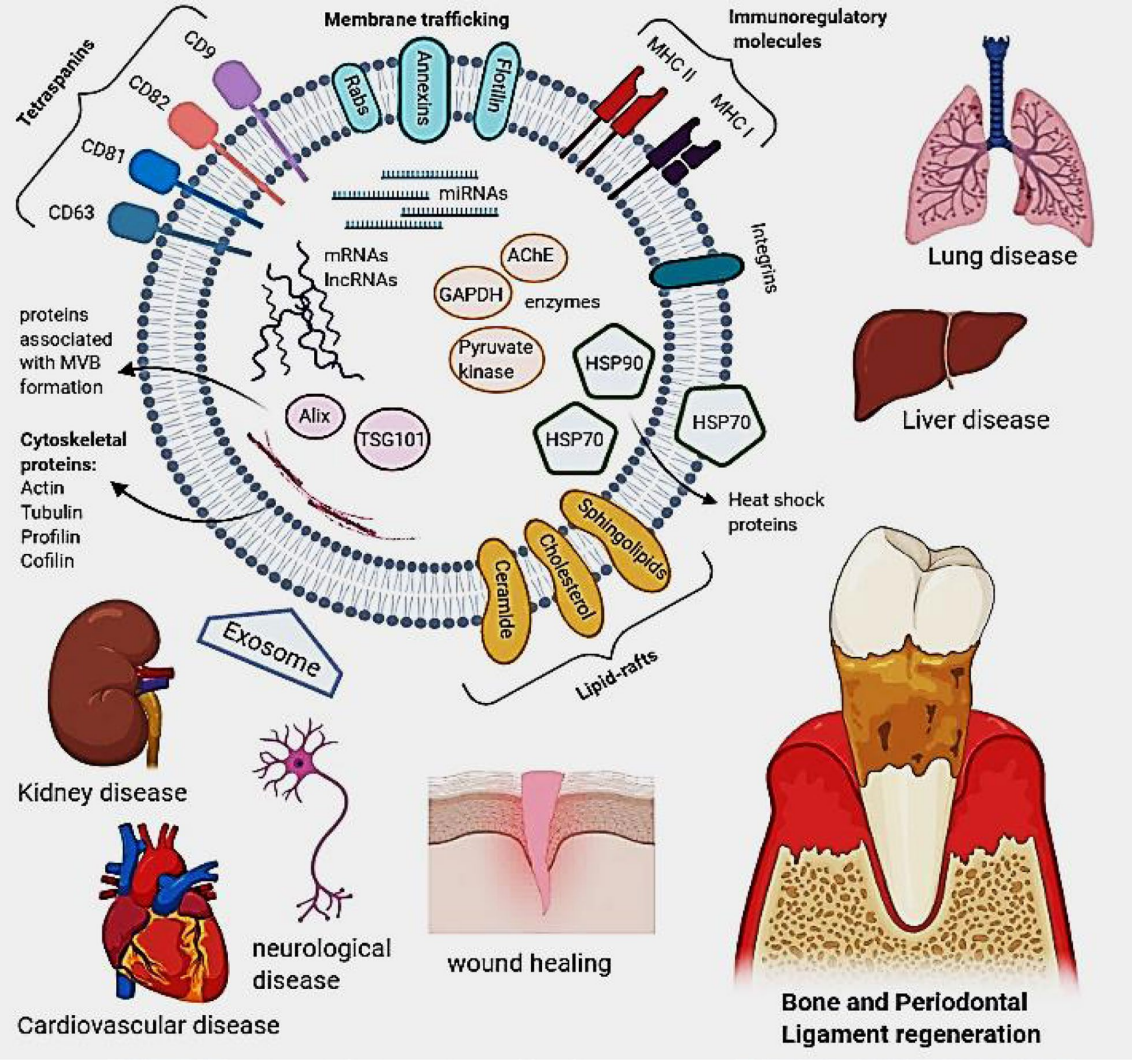
Components and potential applications of extracellular vesicles

Depending on their endosomal origin, EVs/exosomes may contain membrane transport and fusion proteins (Annexins, Rabs, flotillin), tetraspanins (CD9, CD63, CD81, CD82), heat shock proteins (Hsp70, Hsp 90), proteins associated with MVB formation, including Endosomal Sorting Complex Required for Transport (ESCRT) proteins, apoptosis-linked gene 2-interacting protein X (Alix), Tumor Susceptibility Gene 101 (TSG101), transmembrane receptors including MHC molecules and integrins as well as lipid-related proteins and phospholipases [40, 41]. They also contain cytosolic proteins such as cytoskeletal proteins (Actin, Tubulin, Profilin, Cofilin) and various metabolic enzymes (AChE, GAPDH and Pyruvate kinase) [42]. Therefore, it should be taken into consideration that different sources of exosomes may cause a variation in these markers’ expression. Commonly used markers of exosomes identification include tetraspanins, Alix, flotillin, TSG101, and Rab5b [27]. So far, more than 4400 different proteins in addition to the membrane proteins have been recognized as cargo for intercellular communication [43]. Moreover, exosomes contain specific raft-associated lipids such as cholesterol, ceramide, sphingolipids, and phosphoglycerides with long and saturated fatty-acyl chains [44–46]. The genomic molecules such as mRNA, miRNAs, and lncRNAs are mentioned as other exosome components associated with the regulation of gene expression. Exosome miRNA content is specific to the parental cell type and cell condition (e.g., inflammation and hypoxia) (Fig. 2) [47].

EVs are released in body fluids such as blood, semen and urine and may also be isolated from cell culture condition mediums [48–50].

Cell culture media are convenient sources of EVs that can result in a reproducible and high gain of EVs. Because of the high chance of EVs’ contamination in culture media that are hard to distinguish during the isolation process, alternative ways such as EVs-depleted FBS are considered to prevent the influence on the type, cargo, and amount of released EVs [51–56]. Numerous factors affect EVs’ secretion, including oxidative stress, hypoxia, and calcium ions [57]. These vesicles are distinguished by different sets of lipids, functionally active ribonucleic acids (e.g., mRNA, miRNA), and parental cell-derived cytosolic and membrane proteins [58–60]. EV-based therapies are relatively more convenient than cell-based therapeutics. However, identifying the EV separation, storage and retrieval methods which have been shown to significantly alters both the physical and biological properties of EVs, are challenging topics of research, and are yet being extensively studied to help pave the path for a better translation and clinical application of EVs [25, 48, 61, 62]. 

EVs are involved in several biological interactions, such as intercellular communication, transportation of proteins and nucleic acids, tumorigenesis, and metabolism. They may also be used in diagnostic and therapeutic applications in various diseases, as host immune response modulators, and prions carriers [60, 63]. EVs membrane proteins may interact with cell surface and result in intercellular signaling. The mentioned process is done when a vesicles fuses with the target cell membrane via EVs surface proteins such as Alix or TSG101, and tetraspanins such as CD9, CD63, CD81, and CD82 [64, 65]. Also, internalization into a recipient cell may deliver cargo such as proteins and RNA that are active inside the recipient cell [66].

EVs have also been considered as therapeutic nano delivery systems as they have low immunogenicity, a long half-life in circulation, and are capable of penetrating through the brain-blood barrier [67–69]. EVs derived from stem/progenitor cells have the potential to mediate the regenerative responses of MSCs [70, 71]. As studies have revealed, secreted factors (also known as secretome) play a more critical role in tissue regeneration and repair than trans-differentiation capacity of cells [72]. EVs contents usually determine the changes within targeted cells. They have been proven to result in increased proliferation of cells via mitogen-activated protein kinase (MAPK) pathway [73, 74]. Pro-angiogenic properties of EVs from endothelial cells [75], endothelial progenitor cells [76–78], and mesenchymal stem cells (MSCs) [79, 80] are established to be related to their miRNAs contents [75]. Moreover, due to the secretion of anti-inflammatory cytokines and facilitation of M2 macrophage formation, EVs have been identified as the one of the main sources of them [81, 82].

## Regenerative potential of stem cell derived extracellular vesicles

Regenerative medicine involves the transplantation of stem cells into injured organs and tissues, and improving the regenerative potential and function of existing adult stem cells. In the last decades, numerous studies have confirmed the therapeutic potentials of the stem cells [83–85]. Direct usage of living stem cells is however still associated with some complications such as uncontrolled proliferation, tumorigenesis and metagenesis, teratoma formation, and graft-versus-host disease [30, 32]. Besides, the success rate of the treatment may be affected by improper handling methods, storage, and transportation [31]. Consequently, indirect mechanisms such as the application of paracrine secretions, growth factors, and cytokines have been considered as safer alternative treatments. Based on the ability of EVs to mimic stem cell properties, it is assumed that stem cell-derived EVs represent an appropriate therapeutic choice in regenerative medicine [86]. Compared to the direct use of stem cells, EVs could be generated on larger scales. They are smaller, easier to handle, and less expensive. They also have specific targets and have lower potential ethical and legal concerns [87]. These vesicles have high stability and can keep their potency in proper storage conditions for approximately 6 months [88, 89].

They also eliminate the risk of pulmonary embolism formation caused by cell transplantation [90]. EVs have been shown to be able to alter the recipient cells’ functions by providing genetic information that affects their characteristics and paracrine factors and result in tissue regeneration [91]. The evidence has also revealed that the content of EVs is dynamic and largely depends on their cellular origin and physiological status, which needs to be taken into account when used as a therapeutic agent [27].

Another introduced means of application of these vesicles is use of CM in which stem cells were cultured. According to Osugi et al. [92], there are numerous growth factors such as IGF-1, VEGF, and TGF-β1 in serum-free CM from human bone marrow-derived MSCs that can enhance bone regeneration. Positive anti-inflammatory and stimulatory effects on angiogenesis and periodontal and bone regeneration has been reported with application of EVs from stem cell CM [93–95].

Similar to stem cells, it has been observed that the source and origin from which EVs are obtained can change the results of their application. For example, dental pulp MSC-CM showed higher vasculogenesis in vivo and higher antiapoptotic, angiogenic, migration activity, and immunomodulatory effects in vitro in comparison with bone marrow MSC-CM. Human umbilical vein endothelial cells (HUVECs) shape more tube-like structures and cords when in touch with dental pulp MSC-CM, which is known to be the shape of endothelial cells [96]. PDL MSCs, as another dental source of stem cells, have shown a great potential for osseous regeneration [97, 98]. Qin et al. have reported that EVs derived from BMSCs can form more bony structures in the critical-size calvaria bone defects than other cell types [99]. These difference should be considered along with the ease of access and isolation in future craniofacial regenerative studies [100].

## Clinical applications of EVs and their limitations

The therapeutic effects of EVs have been illustrated in various fields, such as cardiovascular, neurological, lung, kidney, and liver diseases. Basu et al. have assessed the effect of current exosomal therapy on neuroregeneration and skin regeneration. They indicated that EVs are more stable and storable than cells. They decrease the risk of aneuploidy and immune rejection caused by in vivo allogeneic administration and might offer a substitutional therapy for different diseases [101].

There are shreds of evidence of EVs and even dental stem cell derived EVs are being successfully utilized in regeneration of other tissues and cure of disease such as the nervous and cardiovascular systems. A previous study reported that SHED-derived EVs are able to improve functional recovery after traumatic brain injury [102]. In addition, Alvarez-Ervitl et al. have shown the amelioration of Alzheimer’s disease by the injection of EVs obtained from modified cells. Also, Ahmed et al. [103] demonstrated that DPSCs might act as a good source for secretome-based therapy of Alzheimer’s disease. It has also been revealed that neurons secrete EVs containing alpha-synuclein and amyloid-beta protein that are, respectively, the indicators of the progression of Parkinson’s and Alzheimer’s diseases [104].

In myocardial infarction in a mouse model, ventricular remolding and the left ventricular ejection fraction were significantly improved after treatment with EVs. This improvement might have been the result of transporting the miR-29 family and IGF-1R from the EVs into the heart [104]. Lee et al. [105] showed that in hypoxia-induced pulmonary hypertension mice, EVs mediated the cytoprotective action of MSCs, which inhibited the disease progression and protected lung from adverse effects of hypertension. In another study, Zhou et al. [74] suggested that human MSC-derived EVs could be exploited as protection against apoptosis and cisplatin-induced renal oxidative stress in vivo and in vitro.

As mentioned, craniofacial regeneration is one of the growing fields of EV application. Furthermore, the therapeutic use of EVs has also gained attention in reconstructing the pulp complex and dentin in recent years. Ivica et al. [106] proposed that pulp-derived EVs, along with a fibrin gel, could be an effective combination for clinical translation on the way to improved cell-free regenerative endodontics. Furthermore, Huang et al. assessed the potential characteristics of EVs from dental pulp stem cells that were cultured in odontogenic differentiation conditions to promote odontogenic differentiation of DPSCs and hMSCs in vitro and in vivo. Their results highlighted the possible role of EVs as biomimetic tools to differentiate stem cells in a lineage-specific manner [107].

Although bone regeneration has been investigated in number of studies in recent years, there are only few available studies that are specifically focusing on EVs application in the regeneration of the tooth periodontium. The available evidence on EVs potential applications in bone and periodontal regenerations will be further discussed.

## Bone regeneration

Bone regeneration via EVs or their CM has been the focus of many studies in recent years (Table 1). As previously mentioned, many researches have indicated that EVs have essential roles in cell-to-cell interaction, organ-to-organ communication and also, can be introduced as a novel signaling mediator in whole-body communications.

**Table 1:** Characteristics of studies conducted on the application of EVs in bone regeneration

Indirect stimulator type	Study mode	Active EV cargo molecules	Key function/targeted genes	References
BMMSC-EVs	In vitro	miR-3084-3p, miR-680, miR-677-3p and miR-5100	RUNX2 and ALP up regulation, as well as matrix mineralization enhancement/activated Wnt/β-catenin	[111]
hMSCs-EVs	In vitro and in vivo (athymic nude mice)	VEGF and BMP2	Induced osteogenic differentiation of naive MSCs and matrix mineralization, increased phosphorylated proteins/up regulation of RUNX2 and Osterix	[112]
Dendritic cells-EVs	In vitro	–	Triggered osteogenic differentiation in MSCs/increased expression of Runx2 and ALP activity	[113]
hASCs-EVs	In vitro and in vivo (mouse calvarial defect model)	miR-34a, let-7a, miR-218	Enhanced bone regeneration/increased expression of RUNX2, ALP, COL1A1	[114]
ASCs-EVs	In vitro	Wnt-3a protein	Induced osteogenic differentiation of HOBs/increased expression of RUNX2, collagen I, osteopontin, and bone sialoprotein	[115]
LPS-activated human monocytes-EVs and CM	In vitro	–	Promoted osteogenic gene expression in MSCs/increased expression of RUNX2 and BMP2	[116]
Mc3t3-EVs	In vitro	Phosphorylated eIF2a	Promoted osteoblastic differentiation	[117]
Cavin-1-PC3-EVs	In vitro and in vivo(NOD/SCID mice)	Proteins and miR-148a	Induced osteoclast differentiation and osteoblast proliferation	[118]
NOS-1 cells-EVs	In vitro	Ca, P, and Zn concentrations	Increased mineral deposition mediated by the activation of ALP and calcium-binding proteins	[119]
hBMSC-EVs	In vitro and in vivo (SD rat calvarial defect model)	miR-196a, miR-27a and miR-206	Promoted osteogenic function/increased expression of ALP, OCN, OPN and RUNX2	[99]
Multiple myeloma (MM) cells-EVs	In vitro	AKT pathway	Modulation of osteoclasts function and differentiation/increased expression of TRAP, MMP9, and CTSK	[120]
Osteoclast-EVs	In vivo (mouse marrow, a model for the in vivo bone microenvironment)	RANK	Regulation of osteoclastogenesis/regulation of 1,25(OH)2 D3-stimulated osteoclast formation	[121]
pMSCs-EVs	In vitro	Actin cytoskeleton, growth hormone, clathrin mediated endocytosis, and VEGF	Stimulated microvascular endothelial cells migration in a concentration and oxygen-dependent manner/promoted vascular network formation	[110]
hiPS-MSC-EVs	In vitro and in vivo (rat model of calvarial bone defects)	PI3K/Akt signaling pathway	Enhanced proliferation and migration ability of hBMSCs and osteogenic differentiation/up-regulation of PDGFA, FGF1/2, FGFR1, COL1A1/2, BCL2L1/down-regulation of PTEN and GSK3β/activation of PI3K/Akt signaling pathway	[122]
Rat bone marrow-derived MSCs-EVs	In vitro and in vivo (nude mice)	–	Enhanced bone regeneration and vessel formation/increased CD-31 positive cells	[123]
BMP2/macrophage-EVs	In vitro	VEGF and BMP2	Increased osteoblastic differentiation and activated autophagy during osteogenic differentiation/enhanced expression of ALP, OPN, IBSP, Runx2, OCN, Col-I, and BMP signaling pathway	[124]
hBMMSCs-CM	In vitro and in vivo (rat calvarial bone defect model)	IGF-1 and VEGF	Enhancement of bone formation and MSC migration into the defect/enhanced expression of OCN and the Runx2 genes	[125]
hBMMSCs-CM	In vitro and in vivo (rat calvarial bone defect model)	IGF-1, VEGF, TGF-B1, and HGF	Increased migration and expression of osteogenic-related genes like ALP, OCN, and RUNX2	[92]
hBMMSCs-CM	In vitro and in vivo (rat calvarial bone defect model)	VEGF	Increased bone formation, and blood vessel formation	[94]
hBMMSCs-CM	In vivo (mouse H-DO model)	MCP-1/-3 and IL-3/-6	Accelerated distraction osteogenesis accelerated new bone callus formation	[126]
hBMMSCs-CM	In vitro and in vivo (rat BRONJ models)	IGF-1, VEGF, angiogenin, IL-6, and M-CSF	Increased bone healing with complete soft tissue coverage/increased expression levels of the Runx2, OCN and VEGF-A	[127]
Hypoxic hDPCs-CM	In vitro and in vivo (mouse DO model)	VEGF-A165 and Ang‐2 protein	Increased *α*-SMA‐positive mature blood vessels and blood vessel formation/promoted new callus formation and upregulation of factor 8 gene	[128]
hFMSCs-CM	In vitro and in vivo (rat DO model)	–	Upregulation of mRNA expression of ALP, OCN, OPN, Osx, and Runx2	[129]
hFMSCs-CM	In vitro and in vivo (nude mice)	Up-regulation of p21and down-regulation of bax and p53	Reduced SA-β-gal expression and activity/enhanced cell proliferation and osteogenic differentiation/activation of sirt1and foxo3a/induced expression of osteogenic genes, including Alp, Runx2 and Rex1/highly expression of COL1A1 and OPG	[130]
hESC-MSCs-EVs	In vivo (critical sized osteochondral defects in a rat model)	–	Restored cartilage and subchondral bone after 12 weeks/high levels of GAG and type II collagen	[131]
hBMMSCs-CM and EVS	In vitro and in vivo (femur fracture model of CD9^−/−^ mice)	High expression of miR-4532, miR‐125b‐5p, and miR‐4516 in EVs. High levels of MCP‐1, MCP‐3, and SDF‐1 in CM	Accelerated fracture healing/increased TRAP-positive cells and cells positive for the vascular marker αSM	[132]
hiPSCs-MSC-EVs	In vitro and in vivo (rat critical size bone defect model)	–	Enhanced cell proliferation and ALP activity/up-regulated mRNA and protein expression of osteoblast-related genes/stimulated bone regeneration and angiogenesis	[133]
uMSCs-EVs	In vivo (rat stabilized femoral fracture model)	-	Enhanced angiogenesis and bone healing processes/enhanced osteogenic differentiation/increased VEGF and HIF-1α expression/enhanced proliferation/migration and tube formation of HUVECs	[134]
DPSCs-EVs	In vitro	–	Enhanced DPSCs viability, migration and mineralization potential/a time-dependent increase of TGF-1 and a TEGDMA concentration-dependent increase of both TGF-1 and FGF-2 in CM	[135]
hGMSCs-CM	In vitro and in vivo (rat calvarial defect model)	–	Induction of new bone formation and osseointegration through expressing or up-regulating genes involved in ossification or regulation of ossification	[136]
hGMSCs-EVs	In vitro and in vivo (rat calvarial defect model)	–	Improved bone healing by showing better osteogenic properties and greater osteogenic inductivity/increased RUNX2 and BMP2/4 mRNA expression/up-regulation of osteoblast differentiation genes/inducing the regulation of adhesion molecules	[137]
hPDLSCs-EVs hPDLSCs-PEI-EVs	In vitro and in vivo (rat calvarial defect model)	–	Up-regulation of osteogenic genes, such as TGFB1, MMP8, TUFT1, TFIP11, BMP2, and BMP4/induced bone regeneration/improved mineralization process and inducing extensive vascular network	[138]
hPDLSCs-CM hPDLSCs-EVs hPDLSCs-PEI-EVs	In vitro and in vivo (rat calvarial defect model)	–	Enhanced osseous regeneration, vascularization, and osseointegration/increased levels of VEGF and VEGFR2	[98]
DMSCs-EVs BMSCs-EVs	In vitro	DSPP, BMP7, DDR2, USP9X, NCOA2, PEG10, LPA	Presence of osteogenic lineage proteins/higher osteogenic differentiation and lower adipogenic differentiation	[139]
Bone marrow interstitial fluid EVs	In vitro and in vivo (mice femoral defect model)	miR-96-5p, miR-182-5p, and miR-183-5p	Suppress osteogenic differentiation, increase cell senescence/decreases Hmox1/suppresses BMSC proliferation and mineralization	[140]
BMSCs-EVs	In vitro and in vivo(intravenous injection in mice)	miR221, miR451a, miR654-3p, miR106b-3p, miR155-5p and miR210-5p	Reversal of growth inhibition, DNA damage and apoptosis on exposure/stimulation of normal murine marrow stem cells to proliferate	[141]
Osteoblast-derived EVs	In vitro	RANKL	Stimulation of RANKL–RANK signaling/facilitation of osteoclast formation	[142]
MC3T3-E1-EVs (mouse preosteoblast cell line)	In vitro and in vivo (mice subcutaneous back defect model)	TRIP-1	Increased matrix mineralization during differentiation/initiation of the calcium phosphate nucleation process/increased expression of Runx2 and alkaline phosphatase	[143]
Osteoclast derived EVs	In vitro and in vivo (mice femoral defect model)	miR-214-3p	Inhibition of osteoblast activity/reduction in bone formation	[144]
Human synovial fluid EVs	In vitro	miR-26a-5p, miR-146a-5p, miR-328-5p, and miR-4654	Regulation of EVs miRNA cargo by estrogen signaling/increased catabolic activity and inflammatory genes/decreased anabolic genes	[145]
Murine-derived BMMSCs-EVs	In vitro and in vivo (mice subcutaneous defect model)	miR-151-5p	Promotion of osteogenic differentiation/reduction of adipogenic differentiation/increased mineralized nodule formation/up-regulation of the Runx2, ALP, and OCN/increased in vivo bone formation/inhibition of IL4Rα expression/down-regulating mTOR pathway activation	[146]
hBMSCs-EVs	In vitro	miR-199b, miR-218, miR148a, miR-135b, and miR-221	Modulatory effect on RNA degradation, mRNA surveillance pathway, Wnt signaling pathway, and RNA transport/potential effect on pathogenesis of osteogenic dysfunction	[147]
hMSCs-EVs	In vitro	miR-31-3p/5p and miR-10b-5p	Increased ALP activity compared with the initial time point/induction of osteogenic differentiation in a stage-dependent manner/enrichment of the pathways endocytosis (hsa04144), regulation of actin cytoskeleton (hsa04810), spliceosome (hsa03040), RNA transport (hsa03013), mRNA surveillance pathway (hsa03015) and protein digestion and absorption (hsa04974)	[148]
hASCs-EVs	In vitro and in vivo (rat calvarial defect model)	miR-375	Improved osteogenic differentiation/inhibition of IGFBP3	[149]
Supernatants of rat bone marrow EPCs derived EVs	In vitro and in vivo (rat unilateral tibial DO model)	miR-126	Accelerated bone regeneration/enhanced mechanical properties/higher vessel density/enhanced proliferation, migration, and angiogenic capacity/down-regulation of SPRED1 and activated Raf/ERK signaling	[150]
hBMSCs-EVs	In vitro and in vivo (rat calvarial defect model)	–	Activation of AKT/mTOR pathway/stimulation of angiogenesis in HUVECs/enhanced bone regeneration and angiogenesis	[151]
EPCs-EVs	In vitro	–	Inhibition of osteoblastic differentiation of BMSCs/inhibition of osteogenic genes expression/increased BMSCs proliferation	[152]
MSCs-CM	In vitro and in vivo (rat calvarial defect model)	–	Enhanced cellular migration/promoted bone regeneration and angiogenesis during early stages	[95]
SHED-EVs	In vitro	Wnt3a and BMP2	Promoted osteogenic differentiation/higher ALP activity/up-regulated osteogenic gene expression (RUNX2, OPN and OCN)	[153]
SMCs-CM PCs-CM	In vitro and in vivo (rat femoral defect model)	Collagen Type I, fibronectin, decorin, and biglycan	Enhanced proliferation, migration and osteogenic differentiation of (BM-MSCs)/accelerated bone formation	[154]
BMSCs-EVs	In vitro and in vivo (mice femoral defect model)	miR-26a	Enhanced expression of Ocn, Runx2 and Alpl/increased ALP activity/promoted matrix mineralization/enhance osteoblastic differentiation/enhanced bone mass/accelerated bone healing	[155]
SMSCs-EVs	In vitro and in vivo (rat model of GC-induced ONFH)	–	Enhanced proliferation and anti-apoptotic responses of BMSCs/inhibition of the decreased osteogenic response caused by MPS injection	[156]
BMSCs-EVs	In vitro and in vivo (rat model of unilateral tibial DO)	–	Increased the ALP activity and calcium mineral deposition/upregulation of ALP, RUNX2, and OCN/mediated through the SDF-1/CXCR4 axis	[157]
hWJ-MSCs-EVs	In vitro and in vivo (rat model of GIONFH)	miR-21	Anti-apoptotic and proliferative effects on MLO-Y4 osteocytes mediated by the PTEN-AKT signaling pathway	[158]
BMSCs-EVs	In vitro and in vivo (rabbit SANFH model)	–	Increased OCN and ALP expression/accelerated trabecula bone regeneration and enhanced proliferation, migration and tube formation of HUVECs through HIF-1α pathway	[159]
hUC-MSC-EVs	In vitro and in vivo (SNFH rats	–	Enhanced callus formation, reduced apoptotic cells, and elevated expressions of CD31, BMP-2, and VEGF	[160]
BMSC-J-EVs	In vitro and in vivo (critical-sized mouse calvarial defect model)	–	Induced osteogenic differentiation with significant increase in ALP and alizarin red staining/upregulation of osteogenic marker (ALP, OSX and RUNX2) expression	[161]
BMSCs-EVs	In vitro and in vivo (ONFH rabbit model)	microRNA-122-5p	Attenuated ONFH development by down-regulating SPRY2 via the RTK/Ras/mitogen-activated protein kinase (MAPK) signaling pathway	[162]
iPS-MSC-Exo	In vitro and in vivo (steroid-induced rat osteonecrosis model)	–	Reduced osteonecrosis, improved bone structure parameters including increased BV/TV, bone surface area over bone volume, Tb⋅Th and Tb⋅N, and enhanced angiogenesis and expression of VEGFR2 and CD31 Enhanced proliferation, migration and tube-forming capacities/activated PI3K/Akt signaling pathway	[163]
hUC-MSCs-EVs	In vitro and in vivo (mouse femoral fracture model)	miR-126	Promoted proliferation, migration, and tube formation of HUVECs/enhanced angiogenesis through a SPRED1/Ras/Erk pathway	[164]
hBM-MSCs-EVs	In vitro and in vivo (rat model of ONFH)	miR-224‐3p	Enhanced angiogenesis by promoting proliferation, migration, and tube formation of HUVECs/ameliorated osteonecrosis, with increased bone viability/increased protein levels of FIP200 and VEGF	[165]
BM-MSCs-EVs	In vitro and in vivo (rat femoral fracture model)	miRNA-128-3p	Increased newly formed callus, BV/TV, and gene expression levels of RUNX2, ALP, and Col I/enhanced osteogenic differentiation through Smad5 inhibition	[166]
hUC-MSCs-EVs	In vitroand in vivo (rat model of disuse osteoporosis)	miR-1263	Improvements in bone histology and structural parameters including increased BV/TV, Tb⋅Th and Tb⋅N, and decreased Tb⋅Sp/reduced apoptosis of bone marrow MSCs with decreased Mob1 and increased YAP expression	[167]
BMMSC-EVs	In vitroand in vivo (rat model of femoral nonunion)	–	Accelerated bone healing with more new bone formation, higher radiographic score and BV/TV/improved proliferation and migration of y HUVECs and MC3T3-E1 cells/increased angiogenesis-related markers (CD31, VEGF, and HIF-1α)/upregulation of osteogenesis markers (OPN and OGN, BMP-2, Smad1, and RUNX2)/overall improved fracture healing score	[168]
hUC-MSCs-EVs	In vivo (rat femoral fracture model)	–	Significant fracture healing with upregulated expression levels of β-catenin and Wnt3a and osteogenic marker genes including Col I, OPN, and RUNX2	[169]
BM-MSCs-EVs	In vitroand in vivo (rat irradiation bone loss model)	–	Reduced oxidative stress, accelerated DNA damage repair, and reduced proliferation inhibition and cell senescence-associate protein expression/activated Wnt/β-catenin pathway	[170]

These naturally occurring nanoparticles include cargos such as genetic materials (mRNA, miRNAs and DNA), proteins and lipids that are able to change the function of targeted cells. If delivered to distinct cells via EVs, mRNAs and miRNAs can alter and regulate gene expression. Not only are EV required in cell-to-cell signaling, but abundant evidence propose that they play a key role in regenerative treatment through their maternal cargos or loaded materials.

There are four target fields in bone regeneration in which EVs have the potential to be utilized: angiogenesis, osteoblast proliferation, intercellular communications, and immunomodulation [108].

Blood vessels are the agents delivering minerals, growth factors, and progenitor cells to the area involved in regenerative activity and help sustain homeostasis. There is evidence suggesting possible angiogenic ability of EVs by improving vessel formation which may lead to stimulation of bone regeneration and growth [109]. It has been revealed that some types of EVs such as placental MSC-derived EVs stimulate endothelial cell proliferation, migration, and tube formation in vitro [110]. Furthermore, there are studies showing angiogenesis improvement in animal models due to EVs injection. Including injection of MSC-derived EVs, which reduced myocardial ischemic injury and improved angiogenesis in the ischemic heart [80].

It has been established that osteoblasts’ primary function is bone formation by producing calcium and phosphate-based minerals. There are shreds of evidence on firming that EVs induce bone regeneration by direct regulation of osteoblast activity and proliferation [171]. Inder et al. [118] demonstrated that prostate cancer cell-derived EVs regulated osteoblast proliferation by 1.5-fold, showing excellent bone affinity. In another in vivo study, bone marrow stromal cell-derived EVs stimulated osteoblastic activity and resulted in an earlier rat calvaria defect healing [99].

EVs role in the bone metabolism is more recognized. For example, the differentiation of both osteoclasts and osteoblasts is activated by osteoclast precursors-derived EVs where osteoblast precursors-derived EVs induce osteoblastic activity [172]. Tan et al. have thoroughly investigated the available literature on bone regeneration using EVs from MCS finding positive therapeutic outcomes in a recent systematic review. They identified several factors influencing the potency of EVs and the outcomes of these regenerative treatments. These factors included the source of EVs, the anatomical origin and developmental age of the tissues for isolation of MSCs dosage/concentration of MSCEVs [173]. Several different bone defect and disease modules have been studied in EV bone regenerative methods with successful alleviation of the pathologic processes involved in bone injury/diseases through improvement of cell migration, survival, proliferation and osteogenic and angiogenic differentiation.

Understanding the underlying pathways of the effect of EV in bone formation has been also investigated. The study of signaling pathways displayed protein cargos of EVs involved in EVs biogenesis and production, internalization and several proteins implicated in osteogenesis. Proteomics analysis of MC3T3-EVs showed the high expression level of associated proteins with the eukaryotic initiation factor-2 pathway. These EVs proteins may have important role in osteoblast differentiation via BMP2. Cui et al., investigated MC3T3-E1 cells by microarray analysis and their findings showed mineralizing osteoblasts (MOBs) EVs contained 457 miRNAs, from which 43 had high expression levels, consisting of several “osteo-miRNAs”known to be expressed in osteoblasts (miR-1192, miR-680, and miR-302a). Analysis of miRNAs expression level in ST2 cells treated with MC3T3-E1 derived EVs detected 91 upregulated (including miR-3084-3p, miR-680, miR-677-3p, and miR-5100) and 182 downregulated miRNAs which cross-talked through the β-catenin gene as a valuable transcription factor in osteoblast differentiation [121, 142].

EV-based intercellular communication between osteoblasts and osteoclasts may be considered as a novel regulator of bone remodeling. Osteoblasts release RANKL containing EVs, which are transferred to osteoclast precursors. It also facilitates osteoclast formation by stimulation of RANKL–RANK signaling. The studies also established the EVs application as a pro-osteogenic approach in bone regenerative field. In addition to their osteogeniccapacity, RANKL protein of UAMS-32P stromal/osteoblastic cell line-EVs can regulate formation of osteoclast cells [121, 142].

Qin et al. [99] showed that miR-196a is the key factor in the regulation of osteoblastic differentiation and the expression of osteogenic genes. Cui et al. [121] have reported that the EVs from mineralizing osteoblasts are capable of entering into ST2 recipient cells in vitro and induce osteoblastic differentiation via the Wnt signaling pathway by affecting Axin1 and βcatenin expression. According to the study by Li et al. aging can change bone metabolism and interfere with it. They showed that miR-214-3p, as a cargo of EVs secreted from osteoclasts, could be associated with less osteoblastic bone formation in elderly patients [144].

Furthermore, some studies have applied EVs through new techniques. For example, Diomede et al. used a three-dimensional printed PLA scaffold and human gingival stem cell-derived EVsto promote bone healing in rat calvaria bone defect [137]. Meanwhile, Zhang et al. showed that EVs combining tricalcium phosphate-modified scaffolds caused an increased osteogenic differentiation in vitro. They also reported promoted osteogenesis in rat calvarial bone defects induced by activating the PI3K/Akt signaling pathway [99]. Together, controlling intercellular communications and signaling pathways by EVs gives us the opportunity of regulating bone metabolism and mineralization.

The mechanisms by which EVs modulate immune system are not yet completely understood; however, they may have the potential to be used as a tolerant therapeutic agent in bone regeneration in immune-competent animals [108]. Ji et al. [174] have demonstrated that DPSC-derived EVs are a potential option that may regulate immune responses. Both immune cells and non-immune cells have the ability to produce EVs to regulate immunity. Antigen-presenting and tumor-derived EVs are the most frequently mentioned immunological EVs. Tumor-derived EVs inhibit macrophage maturation associated with TGF-β. Many researchers have also focused on EVs’ function in inflammation. For example, Ismail et al. [175] reported macrophage-derived EVs enveloping miR-223 regulated macrophage differentiation. As we know, M2 macrophages play an important role in tissue and bone regeneration [176]. Tumor-derived EVs, by contrast, inhibit macrophage maturation associated with TGF-β [177]. Generally, modulation of innate or adaptive immunity by EVs is a potential target for clinical therapeutics in bone regeneration.

## Periodontal regeneration

Routine periodontal treatments can successfully reduce the number of pathogens in a periodontal defect; however, a predictable treatment procedure for reconstructing the lost structures has not been found yet [1]. Some evidence support that MSC-derived EVs can be useful in promoting periodontal ligaments regeneration. As noted, the secretomes of MSCs are known to be responsible for their regenerative effects, containing proteins, lipids, nucleic acid, and trophic factors as growth factors, chemokines, cytokines, hormones, and EVs. Therefore, many studies have started using EVs or their CM as cell-free techniques in periodontal regeneration. Different sources and delivery routes have been used for this purpose (Table 2).

**Table 2:** Characteristics of studies conducted on the application of EVs for periodontal regeneration

Origin/source	Study mode	Active EV cargo molecules	Key function/targeted genes	References
MSCs-EVs	In vitro and in vivo (rat periodontal defect model)	–	Improved periodontal ligament function and promoted regeneration/initiation of prosurvival AKT and ERK signaling in PDL cells/enhanced cell viability	[178]
ADSCs-EVs	In vivo (rat ligature model)	–	Improved periodontal repair and regeneration	[179]
Mobilized-DPSCs-CM	In vitro	–	Greater proliferation and migratory activity of NIH3T3 cells/higher immunomodulatory effects/decreased apoptosis	[180]
PDLSCs-CM	In vivo (rat periodontal defect model)	SerpinE1, MCP-1, TIMP-1, uPA, VEGF, IGFBP6, IGFBP2, PDGFR-β, and IFN-ɣ	Enhanced periodontal regeneration by suppressing the inflammatory response via decrease in TNF-α	[93]
MSCs-CM	In vitro and in vivo (rat periodontal defect model)	IGF-1, VEGF, TGF-β1, HGF	Increased wound-healing and angiogenesis/up-regulation of osteogenetic- and angiogenic-related genes expression/induced periodontal tissue regeneration	[181]
GMSCs-CM PDLSCs-CM	In vitro and in vivo (rat periodontal defect model)	–	Promoted periodontal defect regeneration/lower expression levels of TNF-α and IL-1β/higher IL-10 expression/higher expression levels of BSP-II and Runx2	[182]

Most studies on periodontal regeneration have utilized MSCs CM reporting positive outcomes. Kawai et al. [181] have used Human bone marrow MSCs-CM and reported that it may lead to the enhancement of periodontal tissue regeneration by stimulating angiogenesis and even the mobilization of endogenous MSCs.

Among different sources of MSCs, periodontal ligament stem cells (PDLSCs) are the most commonly studied and potentially considered the most suitable source for periodontal regeneration [183, 184]. They are easily accessible and capable of secreting mineralized structures and can be the best choice for periodontal regeneration due their similar origin. PDLSCs carried by hydroxyapatite/tricalcium phosphate (HA/TCP) were shown to be able to form a cementum/PDL-like structure in vivo [183]. Transplantation of PDLSC-CM has been investigated in some studies and demonstrated considerable new PDL attachment and bone defect regeneration. Nagata et al. investigated the regenerative potential of conditioned mediums (CMs) acquired from cultured periodontal ligament stem cells (PDLSCs) on regenerating periodontal defects models in rats. Their results suggested improved periodontal regeneration and reported a suppression of the inflammatory response caused by TNF-α production as a result of this treatment method [93]. Qiu et al. explored the periodontal tissue regeneration by conditioned media from gingival mesenchymal stem cells (GMSCs) or PDLSCs in rat periodontal defects. Their results showed that similar to PDLSC-CM, GMSC-CM transplantation significantly promoted periodontal defect regeneration in rats. They expressed that this resulted from the regulation of inflammation by MSC-CM and also the facilitation of osteogenic differentiation of bone progenitor cells in the wound region.

EVs isolated from adipose-derived stem cells were used by Mohammed et al. as supplementary treatment to the non-surgical periodontal therapy. They divided 50 rats with ligature-induced periodontitis into four groups, including control, SRP, ADSCs, and exosome group. EVs were locally injected through the pockets by using a plastic syringe after doing a scaling and root planning. They evaluated the progress at different intervals, and the EVs treated group showed the best result with a significantly higher area percentage of newly formed tissues [179].

Chew et al. have also applied EVs derived from hMSCs in regeneration of surgically created periodontal defect in a rat model. They reported that single implantation of collagen sponges has the potential to elevate periodontal regeneration by increasing bone construction and enhancing functional PDL length. They suggested that this could be related to adenosine receptor activation of AKT and ERK signaling pathways, promoting proliferation and migration of PDL cells [178]. MSC-derived EVs have also documented to be able to enhance the repair of osteochondral defects through a reduction in proinflammatory cytokines and an increase in regenerative M2 macrophage counts [185]. Since the activation of pathways that cause bone loss requires an adequate amount of inflammatory factors concentration, the anti-inflammatory characteristic of MSC-derived EVs gained noticeable attention for periodontal regeneration applications [186]. The available evidence indicates that EV based therapies are of valuable therapeutic approaches and have the potential of increasing the success of periodontal regenerative therapies [182]. Further research is still necessary in order to find ideal and standardized sources of EVs, their effective concentration, frequency of treatment and suitable scaffolds or delivery routes used for efficient regeneration of the complex structure of periodontal tissue and further develop their therapeutic applications in this field.

## Future landscapes of EVs applications

Many studies have focused on biomedical EVs applications. Based on their physical functions, EVs of particular cell types have been used as therapeutic mediators in immune therapy, drug delivery, vaccination trials, and regenerative medicine. For example, Xu et al. [187] highlighted the utility of EVs for the development of cancer diagnostics and therapeutics. Mianehsaz et al. [188] reviewed the evidence for EVs from MSCs as a new cell-free therapy method osteoarthritis and joint damage. Synthetic EVs, tunable EVs, and EVs mimetics, as well as EVs designed to overexpress or knockdown signaling pathways associated with pathological conditions, are considered as the next generation of EVs-based products to be studied and developed which may have potential applications in oral and craniofacial diseases [101].

Adequate standards for EVs isolation, manipulation, and characterization need to be defined to reach future progression in the clinical application of EVs. Factors such as the distribution, cargoes, and the purification protocols can manipulate EVs effects [63]. Since EV cargoes depend significantly on their origins, it is essential to profile EVs before clinical applications [189]. There are two commonly used purification procedures, which exploit either repeated ultracentrifugation or ultrafiltration. These techniques provide only a low EVs yield and take a relatively long time. For instance, 5 × 10^6^ myeloma cells can deliver only 5–6 µg of EVs [190, 191]. Achieving good manufacturing practices requires development in EVs isolation techniques. Therefore, developments in EVs studies highly rely on finding novel methods to efficiently isolate them.

It should be noted that delivering EVs in therapeutic dosage to target cells, particularly via systemic injection, may not always be as simple as it looks. Riau et al. proposed the possibility of using the encapsulated form of EVs with biodegradable or highly porous hydrogels. Approaches to encapsulate nanoparticles, like EVs, and instances of possible materials for sustained delivery of the EVs from the stem cells are also main areas of focuses in several studies [192].

After injection, EVs are distributed mostly in the bone, lung, spleen, liver, and kidney. Therefore, it is necessary to assess the clearance and final dosage in the organs [193]. It is still imprecise how to end the biological effects of EVs when the satisfactory outcome is accomplished. Moreover, the half time of EVs applied should be considered to be long enough for achieving the therapeutic aim. Thus, examining EVs in pre-clinical models before moving on to the clinical phase is crucial for their correct translational applications.

## Conclusion and prospects

Several recent studies have been exploring the application of EVs in regenerative medicine. According to these studies, EVs have the potential of regulating immune microenvironment, promoting vascularization, facilitating osteoblasts activity, proliferation and mineralization. Significant development has been made to explore EVs biology, structure, and contents as well as understanding the exact mechanisms by which EVs may alter target cells functions. It is well established that the source of stem cells and their culture conditions affect the functional properties of the secreted EVs. EV-based therapies are considered as novel free-cell therapy approach which is easier to handle and reduces the risk of tumorigenesis, host rejection, and infections associated with direct cell therapy. The available research indicates a great potential for EV application for improvement in success and predictability of bone and periodontal tissue regeneration therapies.

However, we are still facing challenges for ideal clinical application of EVs and further investigations are needed to achieve a protocol for efficient engineering of these nano-bioparticles to maintain exact composition and structure of isolated EVs.

## Data Availability

The datasets generated and analyzed during the current study are available from the corresponding authors on reasonable request by permission of institute and department chairman’s.
